# Exploring the Potential of Nanotechnology in Pediatric Healthcare: Advances, Challenges, and Future Directions

**DOI:** 10.3390/pharmaceutics15061583

**Published:** 2023-05-24

**Authors:** Hossein Omidian, Kwadwo Mfoafo

**Affiliations:** College of Pharmacy, Nova Southeastern University, Fort Lauderdale, FL 33328, USA

**Keywords:** nanotechnology, therapeutic potential, pediatric medicine, drug delivery, disease diagnosis, tissue engineering, nanoparticles

## Abstract

The utilization of nanotechnology has brought about notable advancements in the field of pediatric medicine, providing novel approaches for drug delivery, disease diagnosis, and tissue engineering. Nanotechnology involves the manipulation of materials at the nanoscale, resulting in improved drug effectiveness and decreased toxicity. Numerous nanosystems, including nanoparticles, nanocapsules, and nanotubes, have been explored for their therapeutic potential in addressing pediatric diseases such as HIV, leukemia, and neuroblastoma. Nanotechnology has also shown promise in enhancing disease diagnosis accuracy, drug availability, and overcoming the blood–brain barrier obstacle in treating medulloblastoma. It is important to acknowledge that while nanotechnology offers significant opportunities, there are inherent risks and limitations associated with the use of nanoparticles. This review provides a comprehensive summary of the existing literature on nanotechnology in pediatric medicine, highlighting its potential to revolutionize pediatric healthcare while also recognizing the challenges and limitations that need to be addressed.

## 1. Introduction

In the field of pediatric oncology, nanotechnology has emerged as a tool with significant potential to advance cancer treatment. It provides several advantages, including targeted drug delivery, reduced toxicity, and combined immunotherapy. These features offer promising benefits in the treatment of specific pediatric tumors such as neuroblastoma, retinoblastoma, CNS tumors, and musculoskeletal tumors [1,2]. Nanotechnology-based approaches, including tailored nanocarriers and liposomes, have shown promise in targeted drug delivery with reduced toxicity for pediatric cancers such as acute lymphoblastic leukemia (ALL) and acute myeloid leukemia [3,4,5,6]. Nanovesicles, peptide-functionalized liposomes, and tumor vascular-targeting liposomes have demonstrated effectiveness in neuroblastoma treatment [7,8]. Furthermore, nanomedicines, nanoparticle-based drug delivery systems, and nanotechnological-based miRNA interventions hold promise for addressing neuroblastoma [9,10,11,12,13,14]. Nanotechnology also shows potential in improving outcomes for osteosarcoma treatment through alpha-particle therapy, exosome mimetics, nanocarriers, and targeted drug delivery systems [15,16,17,18,19,20,21,22].

Nanotechnology extends its potential beyond cancer treatment, as it holds promise in pediatric infectious disease management. Nanomedicines enable targeted drug delivery for malaria treatment and leishmaniasis, reducing toxicity while maintaining efficacy [23,24,25]. Nanoparticles have been utilized in bioassays for detecting and controlling schistosomiasis [26]. Additionally, nanocarriers combat antibiotic resistance and enhance the performance of drugs in infectious diseases [27,28]. Nanofabricated biosensors show high sensitivity in detecting bacterial infections, contributing to innovative approaches in combatting pediatric infectious diseases [29]. Furthermore, nanotechnology has contributed in the diagnosis and treatment of tuberculosis (TB) and human immunodeficiency virus (HIV) infections, improving targeted drug delivery, diagnostics, and treatment outcomes [30,31,32,33,34,35,36,37,38,39].

Respiratory and pulmonary diseases also benefit from nanotechnology advancements. Nanoparticle-based technologies have demonstrated effectiveness in preventing biofilm formation and infection in ventilator-associated pneumonia (VAP) [40,41]. Nanotherapeutic approaches show potential in detecting and treating Respiratory Syncytial Virus (RSV) [42,43]. Nanotechnology has improved diagnostic methods for cystic fibrosis and offered pain management solutions [44,45]. These advancements offer opportunities to enhance disease diagnosis, treatment, and patient outcomes in respiratory and pulmonary diseases.

Nanotechnology plays a vital role in addressing critical issues in pediatric environmental health and infectious diseases. It has proven effective in detecting water-borne parasites and pathogens, providing solutions for public health challenges [46,47,48,49]. Nanotechnology offers potential strategies for addressing scorpion envenomation and controlling viral infections [50,51,52]. Coordinated efforts are needed to leverage nanotechnology’s potential in improving public health outcomes and addressing environmental health issues.

In the field of pediatric medicine, nanotechnology offers innovative solutions for the diagnosis and treatment of various conditions. It has shown promise in epilepsy, expanded newborn screening, cardiovascular diseases, neuroinflammation, neurodegenerative diseases, gestational diabetes, bone disorders, mosquito-borne diseases, micronutrient deficiency, vulvovaginitis, and more [53,54,55,56,57,58,59,60,61,62,63,64]. Nanotechnology holds potential for tissue engineering, personalized nanomedicine, scoliosis, respiratory tract disorders, neurosensory diseases, and infections [65,66,67,68,69,70,71,72,73,74,75,76,77].

The impact of nanotechnology extends to various branches of pediatric medicine. In pediatric dentistry, nanotechnology offers promising solutions, particularly in the treatment of occlusal cavities. Nanoparticles incorporated into resin coatings improve wear resistance, prolonging the lifespan of dental restorations [78]. Nanovectors delivering resveratrol oral sprays reduce plaque formation and inflammation, promoting oral health [79]. Furthermore, polysaccharide-based systems offer biocompatibility and drug delivery potential, enhancing treatment outcomes in pediatric dental care [80]. The incorporation of silver nanoparticles in dental sealants creates antibacterial and rechargeable sealants, preventing the onset of dental caries [81]. Additionally, biodegradable magnesium-alloy stents effectively manage pediatric airway obstruction, providing a promising solution for respiratory conditions [82]. While the benefits of nanotechnology in pediatric dentistry are evident, further research is needed to fully understand its benefits and drawbacks [83].

Pediatric dermatology also benefits from the application of nanotechnology, particularly in the management of atopic dermatitis. Nanocarriers improve drug delivery by enhancing solubility and skin permeation, reducing side effects associated with topical treatments [84]. Chitosan nanoparticles, for instance, enhance drug penetration, leading to improved therapeutic outcomes in atopic dermatitis [85,86]. Additionally, polydopamine nanoparticles have shown the ability to inhibit fibrosis in neonatal scleredema, providing new avenues for the treatment of this condition [87]. Nanotechnological carriers hold promise for improving the efficacy and safety of treatments for various pediatric skin disorders, addressing a significant unmet need in pediatric dermatology [84].

Another crucial area where nanotechnology holds potential is pediatric nutrition. It offers innovative solutions for addressing critical issues such as obesity, nutritional deficiencies, and food allergies in pediatric populations [88]. Nanotechnology-based food production can provide more nutritious and low-calorie options, contributing to improved pediatric dietary habits [89]. Furthermore, iron solid lipid nanoparticles offer an alternative to conventional iron supplements, enhancing the bioavailability of this essential nutrient [90]. Nanotechnology-based optical biosensors enhance food safety by enabling the rapid and accurate detection of harmful contaminants [91]. Additionally, nanotechnology-based diagnostics aid in personalized allergen immunotherapy, ensuring safer and more effective management of food allergies in children [92]. Collaborative efforts are crucial for advancing research and ensuring the safety and efficacy of nanotechnology in the field of pediatric nutrition [88].

One area where nanotechnology has made significant contributions is pediatric drug delivery. It has addressed key challenges such as solubility, taste, and stability, improving the effectiveness of drug therapies in children. Biomimetic nanovesicles incorporated into transdermal patches have been developed to enhance the delivery of micronutrients [93]. In situ self-assembly nanoparticles improve the oral delivery of solid dosage forms, increasing drug bioavailability and therapeutic efficacy [94,95]. Folic acid magnetic nanotheranostics have been developed to reduce cardiotoxicity and enhance targeted drug delivery [96]. Nanoparticle-based systems offer ease of administration and enhanced drug delivery across various routes [97]. Nanofibers and nanocapsules provide effective drug delivery approaches, improving the therapeutic outcomes of pediatric medications [98,99,100]. Nanopatch technology offers a needle-free and painless approach to vaccine delivery—particularly relevant for pediatric immunization [101]. Nanocosmeceuticals benefit from nanoformulations, enabling targeted delivery of skincare ingredients [102].

The integration of nanotechnology in pediatric medicine has ushered in a new era of possibilities. However, it is essential to acknowledge that, along with its tremendous potential, nanotechnology also raises concerns regarding potential health risks. Researchers have highlighted the impact of engineered nanoparticles on children’s health, emphasizing the need for thorough investigations into their safety profiles [103]. Studies have specifically examined the neurotoxicity of nanoparticles, shedding light on the importance of understanding their potential risks [104,105]. Carbon nanoparticles and ultrafine particles are areas that require further exploration to determine their impact on pediatric health [106,107,108].

It is crucial to approach nanotechnology with a cautious and responsible mindset. The unique properties of nanoparticles offer biomedical possibilities, but their safe and responsible use must be prioritized [108]. Public understanding of nanotechnology is also paramount, as it empowers individuals to make informed decisions and fosters trust in its applications. Education and awareness campaigns should be implemented to disseminate accurate information about nanotechnology, addressing both its potential benefits and risks. This paper aims to provide an overview of the advances in nanosystems and their potential applications in major pediatric disorders.

### 1.1. Pediatric Diseases

The pediatric population, i.e., children, are at high risk of various diseases and disorders such as malaria [109], iron deficiency [110], traumatic brain injury [111], pediatric cancer [112], respiratory syncytial virus [113], and inflammatory bowel disease [114], to name a few. Malaria is a parasitic disease that affects many children in developing countries, while iron deficiency anemia can lead to fatigue, weakness, and developmental delays. Traumatic brain injury is a significant cause of cognitive, emotional, and behavioral problems, and pediatric cancer can be challenging to treat in children. Respiratory syncytial virus can cause severe respiratory illness in infants and young children, and inflammatory bowel disease causes chronic inflammation in the digestive tract, leading to abdominal pain and diarrhea [113,114].

Other diseases and disorders that affect children include dental biofilm and gingival inflammation, vulvovaginitis, diffuse intrinsic pontine gliomas, neuroblastoma, acute myeloid leukemia, HIV, osteosarcoma chemotherapy, craniosynostosis, retinoblastoma, hereditary angioedema, epilepsy, neurodegenerative diseases, asthma, and liver diseases such as biliary atresia and hepatitis. Vaccines are essential for preventing infectious diseases and protecting the health of children, and routine childhood immunization schedules include vaccines against various diseases such as measles, mumps, rubella, and polio. Effective management and early diagnosis of these diseases are crucial for improving quality of life among children and reducing morbidity and mortality.

### 1.2. Nanosystems

Nanomedicines, a specific class of nanocarriers, have significantly advanced the field of medicine by allowing the targeted and efficient delivery of drugs, imaging agents, and genes to specific cells or tissues in the body [115]. Liposomes and polymeric nanoparticles are examples of nanocarriers that can encapsulate drugs and release them in response to specific triggers, offering controlled and targeted drug delivery [116]. Mesoporous silica nanoparticles, with their high surface area and pore volume, are well-suited for drug delivery, imaging, and biosensing applications [117]. Gold nanoparticles and iron oxide nanoparticles have also been employed for targeted drug delivery and imaging purposes [118]. Biodegradable and CO_2_-derivative cationic polymeric nanoparticles are emerging as promising nanocarriers in drug delivery due to their biocompatibility, biodegradability, and the ease with which their surface charge can be modified for efficient cellular uptake and targeted drug delivery. Lipid-based nanoparticles, such as solid lipid nanoparticles and nanostructured lipid carriers, possess unique properties that make them ideal for drug delivery and imaging applications. Composite scaffolds, which combine nanoparticles with natural or synthetic polymers, have found applications in tissue engineering and regenerative medicine, enabling the repair and regeneration of damaged tissues and organs [119]. Magnetic nanotheranostics are gaining prominence in the detection and treatment of various diseases, including cancer, cardiovascular diseases, and neurodegenerative diseases [120]. The nanopatch is a novel nanocarrier designed for transdermal drug delivery, providing a painless and convenient alternative to traditional injections [121]. Graphene and its derivatives, such as graphene oxide and reduced graphene oxide, possess unique mechanical, electrical, and optical properties that make them suitable for diverse biomedical applications, including drug delivery, imaging, and biosensing [122].

## 2. Pediatric Cancer Treatment and Research

### 2.1. Pediatric Cancers in General

Considerable advancements have been achieved in pediatric oncology, and nanotechnology has emerged as a valuable asset in the fight against cancer. The application of customized nanocarriers for drug delivery has demonstrated promising advantages when treating certain pediatric tumors such as neuroblastoma, retinoblastoma, CNS tumors, and musculoskeletal tumors [1]. Another notable advancement is the use of liposomes as delivery vehicles for anticancer agents in pediatric cancer treatment. This approach has demonstrated improved treatment efficacy while reducing toxic side effects [2].

Nanotechnology-based strategies offer substantial potential for enhancing clinical outcomes in pediatric oncology. These strategies aim to reduce toxicity, achieve targeted delivery, and combine with immunotherapeutic agents. Furthermore, nanotechnology holds promise in various areas, such as prevention, diagnosis, and treatment, encompassing tumor targeting and controlled release [123]. However, the field faces a significant challenge due to the limited availability of nanomedicines for pediatric cancer care [124].

Innovative nanotechnology-based approaches show promise in treating pediatric cancers such as diffuse midline gliomas [125], leukemia [126], osteosarcoma [127], and brain cancers [128]. Nanoparticle-based delivery systems have been found to inhibit tumor cell proliferation and migration in cholesteatoma and pediatric brain tumor cells [129,130]. Nanotechnology-based drug delivery enables the specific targeting of anticancer agents to leukemic cells, thereby reducing toxic side effects [131]. The development of nanotechnology has the potential to improve therapeutic efficiency, drug targeting, reduce toxicity, and mask the bitter taste of drugs, with anticancer drugs being the most frequently encountered therapeutic drug class [132].

For the molecular diagnosis of pediatric sarcomas, NanoString technology has proven to be a reliable approach. It can detect sarcoma-specific fusion transcripts in a single reaction with 100% concordance to RT-PCR [133]. In the proteomic analysis of pediatric ependymoma using high-resolution mass spectrometry, similarities with other pediatric brain tumor entities, such as astrocytomas and medulloblastomas, have been revealed [134]. Table 1 summarizes examples of nanocarrier systems utilized in pediatric medicine.

**Figure 1:**
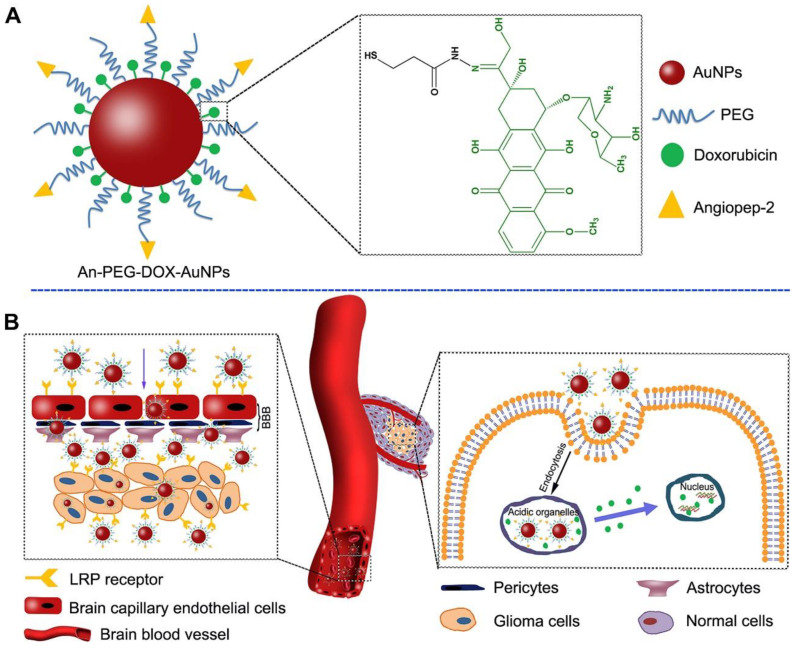
(**A**) Elucidation of the An-PEG-DOX-AuNPs. (**B**) Elucidation of the delivery procedure of An-PEG-DOX-AuNPs. LRP1 receptor could mediate An-PEG-DOX-AuNPs and allow them to penetrate through BBB and target glioma cells, then DOX would be released at the tumor site or in tumor cells and enter into the nuclei to induce tumor cell apoptosis. Printed with permission from [136].

Nanotechnology has shown significant potential in addressing pediatric cancer [135]. Ongoing research in this field is expected to yield innovative and effective treatments for these devastating diseases. Figure 1 illustrates the utilization of two strategies employing gold nanoparticles for delivering doxorubicin (DOX) to gliomas. These strategies involve using Agiopeptide-2 as a targeting polymer and poly(ethylene glycol) (PEG) to evade immune recognition.

### 2.2. Leukemia

Numerous studies have investigated the application of nanotechnology in various types of pediatric leukemia, yielding encouraging results. For instance, the use of CHGNPs (carbon-encapsulated hollow gold nanoparticles) has been shown to selectively induce G1 cell cycle arrest by up-regulating the tumor suppressor protein P27. This advancement provides a cytotoxic drug for the clinical treatment of leukemia [3]. However, the efficacy of lipid-based cubosomal nanoformulations in treating Acute Lymphoblastic Leukemia (ALL) in children has yet to be established. This emphasizes the need for cautious consideration when utilizing nanotechnology to enhance drug efficacy [141].

Gold nanoparticle-based nanocarriers for antileukemic drugs have demonstrated potential in drug delivery, cancer diagnosis, and therapy for ALL. A comprehensive overview of conventional methods and nano-strategies for ALL treatment has highlighted the special focus on gold nanoparticle-based nanocarriers [4]. Similarly, polypeptide-based nanoparticles have shown promising outcomes in depleting CD22DeltaE12 through SiRNA-mediated treatment in B-cell Precursor Lymphoblastic Leukemia [5]. Furthermore, poly(lactide-co-glycolide) (PLGA) nanomedicines loaded with 6-mercaptopurine (6-MP) have exhibited enhanced oral bioavailability and tissue distribution. This has resulted in improved in vitro cytotoxicity of Jurkat cells and prolonged survival time in ALL model mice, offering a promising delivery strategy for clinical translation [142]. The Nessler method, employing ultraviolet-visible spectrophotometry, enables the quantification of PEGylated asparaginase activity in plasma for personalized nanomedicine in clinical settings [143]. Moreover, NanoString nCounter technology has demonstrated robust and cost-effective potential for the diagnosis of B-cell acute lymphoblastic leukemia, boasting high sensitivity and specificity [144].

Polymeric nanoparticles loaded with dexamethasone have been found to enhance therapeutic efficacy, leading to improved quality of life and survival in childhood leukemia [145]. Lastly, the use of siRNA-loaded lipid nanoparticles for LNP-si-LINC01257 treatment has proven to be a safe and effective therapeutic approach for pediatric acute myeloid leukemia [6]. While it is crucial to exercise caution when leveraging nanotechnology to enhance drug efficacy, the potential benefits are evident. The continued exploration and utilization of nanotechnology in the treatment of pediatric cancer holds promise for significant advancements in the field. Table 2 summarizes examples of nanocarrier systems utilized in leukemia treatment.

### 2.3. Neuroblastoma

One highly promising development involves the utilization of nanovesicles coated with GASNGINAYLC peptide [7]. In vitro experiments have revealed that these nanovesicles exhibit exceptional biocompatibility and stability, making them a promising tool for actively targeted nanotherapy in the case of neuroblastoma. Additionally, studies have shown that peptide-functionalized liposomes hold great promise in enhancing tumor-homing properties, inducing tumor apoptosis, and reducing tumor glucose consumption. These unique properties make liposomal nanocarriers a valuable tool for multitargeted treatment of neuroblastoma [8].

**Figure 2:**
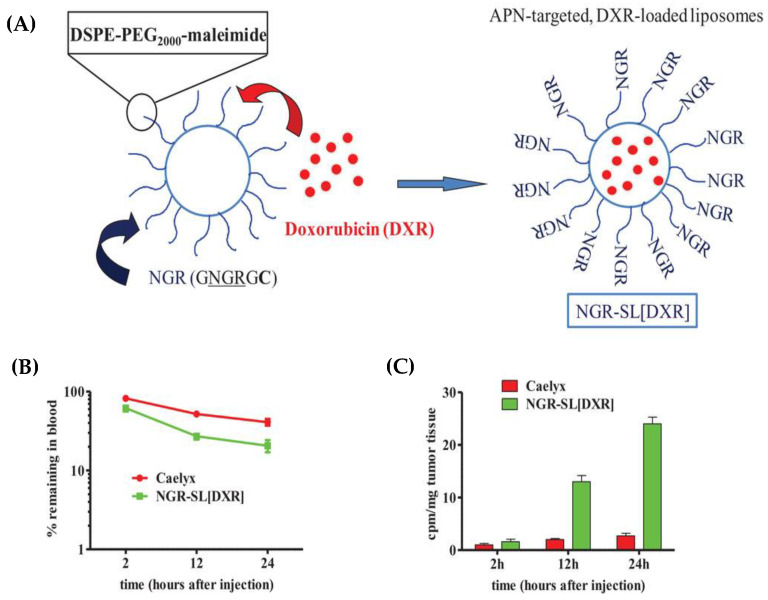
Development of doxorubicin-loaded, tumor vascular-targeting liposomes. (**A**) Schematic representation of the NGR-containing peptide GNGRGGVRSSSRTPSDKYC (called GNGRGC)-targeted, doxorubicin-loaded stealth liposomes (NGR-SL[DXR]). In order to enable coupling of NGR-containing peptide to SL, a cysteine residue (**C**) was added to the peptide C-terminus. (**B**,**C**) Pharmacokinetic profiles and tumor accumulation of NGR-SL[DXR] in NB-bearing mice. Adapted with permission from [8].

Furthermore, recent studies have showcased the development of tumor vascular-targeting liposomes, which allow for the targeted release of drugs. This approach has been successfully tested in mice, demonstrating positive results. Figure 2 visually presents the development of these liposomes and illustrates their release characteristics in mice.

These advancements in nanotechnology offer a potential avenue for the development of effective treatments for pediatric cancer, including neuroblastoma. The use of targeted nanocarriers and liposomes has shown promising results in both laboratory settings and animal studies, suggesting that there is potential for the creation of more efficient treatments for this aggressive form of cancer. Moreover, nanotechnological-based miRNA intervention has demonstrated promise in the therapeutic management of neuroblastoma, addressing challenges related to drug delivery and enhancing therapeutic success [9]. Nanomedicines, such as liposomes and doxorubicin-loaded nanocarriers targeted at nucleolin, have also displayed potential in overcoming the limitations of current diagnostic and therapeutic approaches, offering more effective and targeted options [10,11,12]. Additionally, nanoparticle-based drug delivery systems incorporating etoposide have synergized with alpha v integrin antagonists, improving patient care for high-risk neuroblastoma [13]. The co-assembly of amphiphilic antitumor agents has exhibited better antitumor profiles and controlled release behavior, representing a suitable pre-clinical candidate for childhood cancer therapy in neuroblastoma and osteosarcoma [14].

For anaplastic large cell lymphoma (ALCL), protamine nanomedicine with aptamers, dsDNA/drug payload, and siRNA has the potential to offer cell-selective chemotherapy and oncogene-specific gene therapy by targeting diagnostic biomarkers and therapeutic targets [146]. Additionally, nanomedicines and cell-based therapies are currently being investigated in phase I/II clinical trials for neuroblastoma and medulloblastoma, with the aim of reducing drug toxicity and improving efficacy [147]. Nanomedicine has demonstrated promise in overcoming the limitations of conventional chemotherapy for pediatric neuroblastoma [148]. It offers targeted drug delivery, reduces systemic side effects, and improves pharmacokinetic properties, thereby holding the potential to revolutionize the diagnosis and treatment of childhood cancer. The utilization of nanomedicines enables targeted drug delivery and improved pharmacokinetic properties, leading to a reduction in systemic side effects and the potential to revolutionize the diagnosis and treatment of childhood cancer. Further research in this field is of utmost importance to translate these promising advancements into clinical applications and ultimately improve outcomes for pediatric cancer patients. Table 3 summarizes examples of nanocarrier systems utilized in neuroblastoma treatment.

### 2.4. Osteosarcoma

Osteosarcoma, a challenging form of cancer, is known for its resistance to chemotherapy and lack of effective targeted therapies. Researchers have made notable advancements in the development of diverse nanocarriers, drug delivery systems, and imaging agents. These innovations aim to improve the effectiveness of treatments while minimizing potential side effects. One particularly promising treatment approach involves alpha-particle therapy utilizing (227)Th and (223)Ra, which has demonstrated efficacy in treating multifocal osteosarcoma while exhibiting limited myelotoxicity and high relative biological effectiveness [15]. Additionally, exosome mimetics derived from BMSCs offer a natural platform for nano drug delivery, delivering potent tumor inhibition activity with reduced side effects [16].

Nanocarriers and targeted drug delivery systems also hold potential in overcoming drug resistance and minimizing side effects [17,18]. For instance, lipid nanoparticles loaded with edelfosine have been found to inhibit cell growth in vitro and prevent metastasis in vivo [19]. Furthermore, self-stabilized hyaluronate nanogels co-delivering doxorubicin and cisplatin have demonstrated enhanced antitumor efficacy and reduced side effects [20].

The use of near-infrared imaging and multifunctional graphene-based nano-drug delivery systems has exhibited highly selective anticancer efficiency by targeting mitochondria, offering synergistic phototherapy for drug-resistant osteosarcoma [21]. Moreover, IL-11Ralpha-targeted nanoparticles have shown superior efficacy in treating osteosarcoma by specifically targeting tumor cells. These nanoparticles have demonstrated strong anti-tumor effects in orthotopic and relapsed osteosarcoma models, as well as patient-derived osteosarcoma xenografts [22].

**Figure 3:**
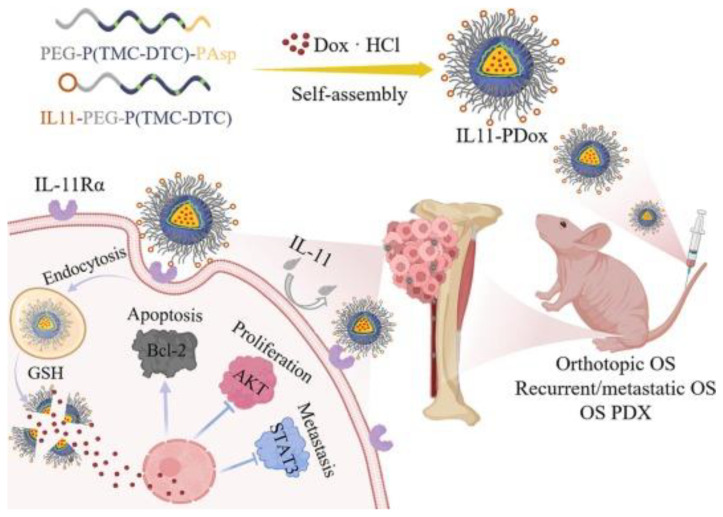
Illustration of fabrication of IL-11Rα-targeting polymersomal Dox (IL11-PDox) and strong inhibition of growth, recurrence, and metastasis of malignant osteosarcoma. Adapted with permission from [22].

Figure 3 provides a schematic representation of the fabrication of IL-11Rα-targeting polymersomal Dox and its mechanism of inhibiting the growth, recurrence, and metastasis of malignant osteosarcoma. Additionally, Table 4 summarizes examples of nanocarrier systems employed in osteosarcoma treatment.

### 2.5. Other Cancers and Cancer-Related Topics

One of the significant challenges in treating pediatric brain tumors, including brain tumors in children, is the blood–brain barrier, which limits effective drug delivery. However, nanotechnology has demonstrated potential with respect to overcoming this obstacle by facilitating drug delivery across the blood–brain barrier [154,155,156,157,158,159,160]. Moreover, nanotechnology offers the ability to selectively target pediatric brain tumors and enhance the bioavailability of phytoconstituents for treating medulloblastoma [158,159]. In the field of ophthalmology, photodynamic therapy utilizing mesoporous silica nanoparticles holds promise for the treatment of retinoblastoma [161]. In regenerative medicine and cancer treatment, nanomedicine-based therapies that combine stem cells with drug delivery systems have shown great potential for achieving improved results [162]. Additionally, gold nanoparticles have been studied for tumor diagnostics through imaging and as delivery devices for targeted therapy in adenoid cystic carcinoma [163]. These examples exemplify the potential of nanotechnology to overcome delivery challenges and provide effective treatments for various pediatric cancers, including osteosarcoma. Table 5 shows additional examples of nanocarrier systems utilized in the treatment of other pediatric cancers or cancer-related disorders.

## 3. Infectious Disease Management and Treatment

### 3.1. Antimalarial/Antibacterial Treatment

In the context of malaria treatment, nanomedicines have proven to be effective tools for targeted drug delivery against the disease [23]. Nanotechnology offers the capability to design strategies that specifically target drug molecules to different stages of the malaria parasite’s life cycle, address drug-resistant strains, and enhance vaccine effectiveness [24].

For the treatment of leishmaniasis, nanotechnology-based drug delivery systems have been developed to minimize toxicity while maintaining therapeutic efficacy [25]. Moreover, nanotechnology presents innovative solutions for administering drugs to pediatric patients affected by malaria, leishmaniasis, toxoplasmosis, and schistosomiasis [171].

Nanoparticles have also been utilized in bioassays for the detection and control of schistosomiasis, offering improved sensitivity, speed, and convenience [26]. In the case of Praziquantel (PZQ), nanocarriers have been developed to overcome the limitations of its low solubility and bioavailability, thereby enhancing its performance [27]. Nanotechnology has also been employed to combat antibiotic resistance by augmenting the antimicrobial efficacy of ceftriaxone against Gram-positive and Gram-negative bacteria using chitosan nanoparticles, providing an alternative to traditional antibiotics [28].

The application of nanotechnology extends to the treatment of pediatric infectious diseases and solid tumors, with its scope ranging from in vitro studies to clinical trials [172]. Additionally, a biosensor employing nano-fabricated structures and anti-E. coli antibodies has exhibited high sensitivity for clinical use in the detection of bacterial infections in human kidneys [29]. Table 6 provides a summary of examples of nanocarrier systems employed in the treatment of pediatric malaria and other bacterial diseases.

### 3.2. COVID-19

Recent studies have showcased the potential of nanotechnology-based diagnostic methods in accurately detecting extracellular vesicles carrying SARS-CoV-2 RNA in plasma, presenting a promising alternative to traditional respiratory RNA level detection approaches. These advancements, encompassing CRISPR-based and optical-based sensing systems, hold significant promise for the development of efficient and rapid diagnostic techniques for COVID-19 [173,174].

Nanotechnology extends beyond diagnostics, offering promising prospects for treatment modalities, vaccination strategies, and the potential integration of artificial intelligence in the field of infectious diseases, including COVID-19 [175]. Moreover, the potential of electrochemical nano-biosensors, utilizing nanomaterials for signal amplification, has been demonstrated in detecting harmful DNA mutations in newborn infants with high sensitivity, a wide dynamic range, and exceptional specificity. This presents a valuable tool for newborn screening purposes [176]. Table 7 provides an overview of examples of nanocarrier systems employed in the treatment of COVID-19.

Overall, the integration of nanotechnology into the realm of infectious diseases holds potential for the development of innovative and effective diagnostic and treatment strategies. Ongoing research and development efforts in this field are anticipated to yield breakthroughs in the battle against infectious diseases, including malaria, bacterial infections, and viral infections such as SARS-CoV-2. The promising results reported in existing studies indicate that nanotechnology will play a significant role in the future of healthcare, revolutionizing the detection and treatment of various infectious diseases [173,174,175,176].

### 3.3. TB and HIV

Nanomedicine has paved the way for new treatment possibilities, including the delivery of antimicrobial host defense peptides, which have shown to enhance therapeutic effectiveness and reduce resistance in TB and HIV infections [30]. Nanotechnology-based diagnostic methods have exhibited potential in accurately diagnosing HIV in infants—a challenging population to detect [31]. Furthermore, the formulation of antiretroviral drugs with nanotechnology has improved their bioavailability, reduced dosage requirements, and enhanced treatment outcomes in HIV patients, particularly among pediatric populations [32,33,34].

Regarding TB, nanotechnology-based antigen testing and polymeric micelles have demonstrated high diagnostic accuracy and increased oral bioavailability of rifampicin, respectively, enabling early detection and effective treatment [35,36]. Various nanotechnology-based strategies have been proposed to develop more effective and patient-compliant medicines for TB treatment, including targeting infection reservoirs and overcoming drug resistance [37]. A child-friendly nanoemulsion containing rifampicin has shown promise in increasing drug bioavailability and reducing treatment failure in pediatric TB patients [39]. With increased institutional support, the integration of nanomedicine and genomic research holds the potential to achieve TB elimination by 2050 [38]. Table 8 provides a summary of examples of nanocarrier systems used in the treatment of pediatric TB and HIV.

### 3.4. Respiratory and Pulmonary Diseases

Nanoparticles have proven effective in preventing biofilm formation and colonization on endotracheal tubes in pediatric patients with VAP, thereby reducing the risk of infection [40]. Moreover, nanomodified endotracheal tubes have shown substantial reductions in the growth of *P. aeruginosa*, effectively combating VAP [41]. Nanotechnology-based therapeutic approaches hold promise for the detection and treatment of RSV with maximum efficacy and minimal side effects [42]. Gold nanorods, for instance, have demonstrated the potential to inhibit RSV by activating the immune response, making them a potential antiviral agent against RSV [43]. In cystic fibrosis, nanotechnology has facilitated the development of the Nanoduct sweat test system, which offers improved ease of use and higher diagnostic success rates in newborns compared to the Macroduct/Gibson and Cooke methods [44]. Furthermore, nanotechnology-based approaches have shown promise in managing the pain associated with cystic fibrosis, a common affliction for CF patients [45]. Table 9 provides an overview of nanocarrier systems utilized in the treatment of pediatric respiratory and pulmonary diseases.

### 3.5. Environmental Health and Infectious Diseases

In recent years, the application of nanotechnology in the field of environmental health and infectious diseases has garnered significant attention from scientists and researchers [46]. This emerging field has demonstrated remarkable potential in the detection and treatment of various diseases. However, it is important to acknowledge the associated risks. Studies using human placental perfusion models have indicated that nanoparticles have the ability to cross the placental barrier, raising concerns about potential risks to developing fetuses [46]. Nevertheless, nanotechnology has shown effectiveness in detecting water-borne parasites and mitigating biological contamination in drinking water, especially in areas with inadequate sanitation facilities in developing countries [47,48]. Nanotechnology-based assays and nanodevices have also exhibited promise in the identification of water-borne pathogens, which is crucial for safeguarding public health [49]. Given the escalating production of environmental pollutants and the health threats posed by climate change, concerted efforts are necessary to tackle these environmental health challenges [178]. Moreover, nanotechnology offers potential solutions for combatting the lethal effects of scorpion envenomation and presents strategies for the treatment and control of viral infections [50,51]. While PCR-based assays currently remain the gold standard for the detection of certain viruses, researchers are actively exploring the advantages of nanotechnology and advanced genetic platforms for therapeutic interventions [52]. Table 10 provides an overview of nanocarrier systems employed in the treatment of pediatric infectious diseases related to the environment.

## 4. Nanotechnology in Other Pediatric Related Areas

### 4.1. Medical Disorders

Liposome nanotechnology has enabled sustained delivery systems of glucocorticoids for epilepsy treatment, enhancing therapeutic efficacy [53]. The integration of mass spectrometry, genomics advancements, and nanotechnology has facilitated cost-effective expanded newborn screening, enabling the detection of a wider range of disorders in inborn errors of metabolism [54].

Saliva has shown potential as a diagnostic fluid for noninvasive and cost-effective detection of cardiovascular diseases and cancers, although there are still clinical challenges to overcome [55]. Core-cross-linked nanoparticles have demonstrated the ability to reduce neuroinflammation and limit secondary injury spread in a mouse model of traumatic brain injury [56]. Nanotechnology has also improved bioavailability and reduced side effects in the treatment of neurodegenerative diseases such as Parkinson’s, Alzheimer’s, multiple sclerosis, amyotrophic lateral sclerosis, Huntington’s, and Wilson’s diseases [57]. Additionally, nanotechnology-based approaches and biosensors hold promise for the high-performance diagnosis of gestational diabetes and jaundice, providing important monitoring tools during pregnancy [58].

Nanotechnology has also shown potential in aiding the diagnosis and treatment of pediatric bone conditions such as type III Osteogenesis Imperfecta, providing insights for effective management [59,60]. Nanofiltered C1 Esterase Inhibitor has been proven to effectively prevent hereditary angioedema attacks during dental, medical, or surgical procedures, with no reported adverse events [61]. Furthermore, the use of polymeric nanocapsules containing geraniol and icaridin has shown efficacy and safety in combating the Aedes aegypti mosquito—a major disease transmitter [62]. Nanoparticle coating techniques have demonstrated the ability to enhance the bioavailability of vitamin B(12) in food crops, addressing the risk of micronutrient deficiency and associated health issues [63]. Notably, the management of vulvovaginitis in girls has benefited from the use of bioyoghurt, probiotics, and petroleum jelly [64].

The integration of nanotechnology with pure sciences and the technologies of the fourth industrial revolution holds the potential for significant advancements in pediatric healthcare, including the development of nano-doctors that could eliminate the need for invasive surgeries and revolutionize diagnostics and therapeutics [65,66]. Albumin and liposome nanoparticles have also shown promise in the treatment of pediatric diseases [67]. Moreover, a thermosensitive liposome formulation combined with mild hyperthermia has improved the therapeutic index of vinorelbine for the treatment of Rhabdomyosarcoma [140]. Advanced microengraving technology has efficiently identified antigen-specific T-cell responses for T-cell immunology [68].

Nanotechnology-based delivery systems offer a potential solution to address the lack of pharmacokinetic data for pediatric drug development [69]. Additionally, these delivery strategies show promise in effectively treating traumatic brain injury (TBI) by bypassing biological barriers and enhancing target engagement [70]. Furthermore, nanomedicine holds potential with regard to developing urinary bladders for children with congenital bladder dysfunction, regenerating kidney, bladder, and urethra tissues using stem cell therapies, myoblasts, fibroblasts, and three-dimensional stem cell-derived organoids, addressing pediatric urological conditions [71]. Nanomedicine-based therapies have also demonstrated promise in managing pregnancy complications, improving outcomes for both mothers and unborn children while reducing the need for emergency caesarean sections [72]. Personalized nanomedicine has shown potential in the treatment of cerebral palsy using gold nanoparticles coated with targeted dendrimers in conjunction with CT imaging and transcranial magnetic stimulation, leading to improved motor function in affected children [73]. Nanomedicine and stem cell therapy offer possibilities for diagnosing and treating high-risk factors associated with cerebral palsy, such as prematurity and low birth weight [74]. Furthermore, nanofiltered human C1 inhibitor concentrate (C1-INH NF) has proven to be a safe and effective replacement for deficient plasma C1 inhibitor levels, reducing the incidence of angioedema attacks in hereditary angioedema [75]. Nanotechnology has also demonstrated its utility in scoliosis management, with ultra-low-dose full-spine protocols providing reliable and repeatable measurements of the Cobb angle with minimal radiation exposure [76]. Moreover, diagnostic and therapeutic applications of nanotechnology show promise in fetal, neonatal, and pediatric diseases affecting the respiratory tract, neurosensory system, and infectious conditions. However, the acquisition of further data is necessary to ascertain their safety and efficacy [77].

Table 11 provides a summary of examples of nanocarrier systems utilized in various medical treatments. In conclusion, nanotechnology-based treatments hold great potential for addressing a wide range of pediatric conditions, including tissue engineering, stem cell therapy, and personalized nanomedicine. These innovative approaches offer potential solutions for the development of new scaffolds for pediatric urological conditions, the management of pregnancy complications, cerebral palsy, hereditary angioedema, scoliosis, and other diseases affecting the respiratory tract, neurosensory system, and infectious conditions. However, further research is necessary to validate their efficacy and ensure their safety.

### 4.2. Dental Disorders

Nanotechnology has emerged as a promising field in pediatric dentistry, particularly in the treatment of occlusal cavities in children. One notable application of nanotechnology is the enhancement of wear resistance in dental materials through the use of resin coatings containing nanoparticles. This approach offers a more durable solution for young patients, as evidenced by studies demonstrating improved wear resistance [78].

Another development in pediatric dentistry is the use of nanovectors to deliver oral sprays containing resveratrol. These sprays have shown significant efficacy in reducing dental plaque and gingival inflammation in early childhood [79]. However, it is crucial to conduct further research to fully understand the potential drawbacks of nanoparticles in pediatric dentistry [83].

It is important to consider recent advancements in nanotechnology alongside the insights from the 2002 Pediatric Restorative Dentistry Consensus Conference [182]. Polysaccharide-based micro- and nano-sized drug delivery systems hold great promise for drug administration in pediatric dentistry due to their biocompatibility, biotolerance, biodegradability, and low toxicity [80]. Additionally, silver nanoparticles (AgNPs) exhibit potent antimicrobial properties and can potentially be incorporated into dental materials to improve their mechanical and antibacterial characteristics, potentially enhancing oral health outcomes in pediatric patients [81]. The integration of nanoparticles of amorphous calcium phosphate into dental sealants can also provide antibacterial and rechargeable sealants with desirable properties and elevated levels of calcium and phosphate ion release [183].

Furthermore, biodegradable airway stents made from magnesium alloys have demonstrated feasibility and efficacy in managing pediatric laryngotracheal stenosis. These stents offer a less invasive and more effective approach to managing pediatric airway obstruction [82]. Table 12 provides examples of nanocarrier systems utilized in the treatment of pediatric dental diseases.

### 4.3. Dermatological Disorders

Among the various skin conditions affecting children, atopic dermatitis has received significant attention in the development of nanocarrier-based drug delivery systems. These nanocarriers have demonstrated high efficacy in enhancing drug solubility, thermodynamic activity, and skin permeation, thereby reducing side effects and improving the management of atopic dermatitis [84]. Studies have specifically highlighted the effectiveness of nanomaterials such as chitosan nanoparticles in enhancing drug penetration and efficacy for the treatment of atopic dermatitis [85,86]. Furthermore, in the case of neonatal scleredema, the use of polydopamine nanoparticles coated with stem cell membrane fragments and doxorubicin has shown inhibitory effects on fibrosis, suggesting the potential of nanotechnology in treating this condition [87]. Although further advancements are necessary to fully exploit the potential of nanocarriers for anti-acne drugs as they hold significant promise in enhancing the effectiveness and safety of such treatments [184]. Table 13 provides a summary of examples highlighting nanocarrier systems employed in the treatment of pediatric dermatologic diseases.

### 4.4. Nanotechnology in Pediatric Nutrition

The obesity epidemic, in particular, requires multidisciplinary collaborations between engineers, physical scientists, and nutrition experts to develop innovative technologies that support therapeutic advancements and promote behavioral changes [88]. By utilizing nanotechnology-based food production, it becomes possible to create more nutritious and lower-calorie food options that can help address nutritional deficiencies, obesity, and type 2 diabetes [89]. To combat common issues such as iron deficiency, researchers have developed iron solid lipid nanoparticles as an alternative to commercially available supplements, overcoming certain limitations [90]. Additionally, green-synthesized iron oxide nanoparticles show promise as antianemic preparations, although safety concerns must be thoroughly addressed [186].

Furthermore, nanotechnology-based optical biosensors, such as gold nanoclusters, have significant potential in ensuring food safety by detecting harmful pathogens and chemical substances [91]. In the context of food allergies and anaphylaxis, nanotechnology-based diagnostic methods can enable personalized allergen immunotherapy and avoidance diets to effectively manage the increasing prevalence of food allergies [92]. To drive progress in this field, collaborative efforts among food producers, policy makers, and health authorities are necessary to address safety concerns and provide adequate funding for research. Table 14 provides a summary of examples illustrating the utilization of nanocarrier systems in pediatric nutrition.

### 4.5. Drug Delivery

Nanotechnology has played a pivotal role in advancing pediatric drug delivery, addressing critical issues such as poor water solubility, taste-masking, and drug stability. One notable application is the use of biomimetic nanovesicles incorporated into transdermal patches, which have demonstrated promising outcomes in enhancing micronutrient delivery for infants and mothers [93]. In situ self-assembly nanoparticle technology has emerged as a potent platform for drug delivery, improving the stability, palatability, and bioavailability of pediatric oral solid dosage forms [94,95]. Folic acid magnetic nanotheranostics hold the potential for safe and effective drug delivery, reducing cardiotoxicity and enhancing drug uptake [96]. Additionally, nanoparticle-based drug delivery systems have enabled the production of easily consumable spheroids, contributing to improved patient compliance [97]. Nanofibers composed of polycaprolactone and polyvinyl alcohol have shown promise in oromucosal drug delivery approaches for children and elderly patients [98,99]. The utilization of spironolactone-loaded nanocapsules has demonstrated a favorable outcome, with stable nanocapsules and high encapsulation efficiency, allowing for lower volumes of liquid preparation [100]. Nanopatch technology has garnered significant acceptability for vaccine delivery, particularly in low and middle-income countries [101]. Furthermore, nanoformulations are necessary for nanocosmeceuticals containing gallic acid and its derivatives due to their poor water solubility and biodegradability [102]. Examples of nanocarrier systems in drug delivery are summarized in Table 15.

**Figure 4:**
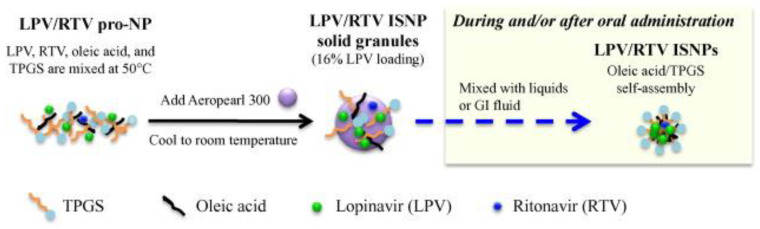
Preparation of LPV/RTV ISNP granules and formation of LPV/RTV ISNPs. LPV and RTV completely dissolve in oleic acid and TPGS to form the pro-NP that is coated on the surface of Aeropearl 300. Once LPV/RTV ISNP solid granules are introduced into liquids (e.g., water during administration as a sachet or the fluid in the GI tract after administration as a sprinkle), oleic acid and TPGS form the ISNPs by a self-assembly process; meanwhile, LPV and RTV are entrapped into the ISNPs during the NP formation. Adapted with permission from [95].

Nanotechnology-based drug delivery systems present novel approaches to enhance pediatric drug administration, minimize adverse effects, and improve the efficacy of existing therapies. Notably, the development of fixed-dose combinations, such as lopinavir/ritonavir, utilizing innovative in situ self-assembly nanoparticle technology, exemplifies the immense potential of nanotechnology in drug delivery (Figure 4). However, further research and development efforts are essential to fully harness the capabilities of nanotechnology in pediatric drug delivery.

## 5. Other Pediatric Applications

Highly sensitive assays using LC-MS/MS technology have identified melatonin and N-acetylserotonin as potential biomarkers for sleep-related disorders, providing valuable insights for diagnosis and treatment [188]. In audiology, nanotechnology research has shown promise in developing advanced sound and hearing implants, offering a potential breakthrough for individuals with profound deafness [189]. Moreover, the presence of metallic particles in human tonsil tissue and amniotic fluid has raised intriguing possibilities regarding their role in disease causation and emerging nanopathology [190].

The concern over nanoparticle exposure has led to investigations into resuspension rates, revealing variations depending on the product, flooring, and resuspension force. Products containing copper, silver, and zinc nanomaterials exhibited higher rates, highlighting the importance of further research and regulation in this area [191]. Advancements in drug delivery systems and nanomedicines hold promise for treating degenerative ocular diseases that manifest in childhood, offering the potential to significantly enhance the quality of life for affected pediatric patients [192]. Similarly, the use of nanocarrier-mediated drug delivery has garnered support and is clinically recommended for the treatment of atopic dermatitis [185].

Studies exploring microbial interactions have unveiled the strong binding of Streptococcus mutans-derived exoenzyme GtfB to Candida albicans, shedding light on the modulatory role of this interaction [193]. Additionally, the implementation of a nano-selenium reactive barrier approach has shown success in suppressing mercury release from compact fluorescent lamps, aiding in the identification of mercury contamination sources and achieving significant reductions in exposure scenarios [194]. A gold nanoparticle-based dynamic light scattering (DLS) probe has demonstrated potential for on-site monitoring of lead (Pb) levels in various samples, detecting concentrations as low as 100 ppt, which surpasses the EPA standard limit by nearly two orders of magnitude [195]. Table 16 provides an overview of nanocarrier systems employed in other areas of pediatric health.

## 6. Potential Risks and Health Effects

Despite its potentially transformational role, it is crucial to carefully assess and address the potential risks associated with this rapidly advancing technology. Several studies have shed light on the potential health implications of nanotechnology, emphasizing the need for caution. For instance, one study [103] investigated the impact of engineered nanoparticles on children’s health, while others [104,105] focused on the neurotoxicity of nanoparticles. Additionally, the health effects of carbon nanoparticles and ultrafine particles warrant further investigation [106,107,108].

Although graphene is generally considered a safer alternative to carbon nanotubes, it is still essential to implement specific safety protocols when working with any type of nanomaterial. Studies have shown that nanoparticles can impose metabolic burden, oxidative stress, and potentially alter milk composition in breastfeeding systems, indicating potential risks [196]. Moreover, children are particularly vulnerable to the potential hazards associated with engineered nanoparticles, necessitating focused research on exposure levels and health consequences [197].

There is a significant lack of public understanding about nanotechnology, especially among middle-school children, despite its profound impact on various industries and society as a whole [198]. Therefore, it is crucial to educate the public about both the potential risks and benefits of nanotechnology. While research is needed to comprehend the disparities between children and adults in terms of harmful effects induced by exposure to ultrafine particulate matter, the unique physicochemical properties of nanoparticles offer promising opportunities for biomedical applications. Hence, it is essential to explore the potential benefits of nanoparticles within the field of nanotechnology [108]. Table 17 provides an overview of potential health risks associated with nanosystems in pediatric nanomedicine.

In conclusion, while nanotechnology holds tremendous promise, it is imperative to carefully evaluate and mitigate its potential risks to ensure its safe and responsible utilization.

## 7. Conclusions

In conclusion, nanotechnology has been identified as a potential tool in the field of pediatric medicine, offering new possibilities for the diagnosis and treatment of various conditions. Its use in pediatric oncology shows promise, as it allows for targeted drug delivery, reduced toxicity, and combined immunotherapy, which may have benefits in treating specific pediatric tumors. Nanotechnology-based approaches have shown potential in delivering drugs directly to the affected areas in pediatric cancers such as leukemia and neuroblastoma. Additionally, nanotechnology has potential applications beyond cancer treatment, including the management of pediatric infectious diseases, respiratory and pulmonary conditions, and environmental health concerns. The integration of nanotechnology has led to advancements in drug delivery, diagnostics, and treatment outcomes. However, it is important to approach nanotechnology carefully and ensure the responsible use of nanoparticles. Thorough research is needed to understand their safety profiles, particularly in relation to potential health risks in children.

## Figures and Tables

**Table 1:** Nanosystems in general pediatric cancer medicine.

Disease/Condition	Key Findings	Ref.
Pediatric cancer	Tailored nanocarriers for drug delivery—show potential benefits for specific types of pediatric tumors, including neuroblastoma, retinoblastoma, CNS tumors, and musculoskeletal tumors.	[1]
	Liposomes—have potential as delivery vehicles for anticancer agents in pediatric cancer treatment, improves efficacy and reduces toxic side effects, summary of potential strengths and technical difficulties.	[2]
	Nanotechnology-based strategies—show promise in improving clinical outcomes by decreasing toxicity, achieving targeted delivery, and combining with immunotherapeutic agents.	[135]
	Nanotechnology—promising applications in prevention, diagnosis, and treatment, including tumor targeting and controlled release.	[123]
Childhood Cancers	Nanoparticle-based compounds—nanotechnology can reduce toxicity and improve the therapeutic index of cytostatic drugs for childhood cancers, but the lack of nanomedicines for pediatric cancer care is a major challenge. This review article provides an overview of conventional methods and nano-strategies for childhood cancer treatment.	[124]
Pediatric diffuse midline gliomas (DMGs)	Nanotechnology-based approaches—show potential through targeting epigenetic alterations, identifying new molecular pathways, exploring immunotherapy, and innovative delivery.	[125]
Pediatric leukemia	Lipoprotein-based drug delivery systems—have potential to enhance therapy, improve bioavailability, and increase efficacy of anticancer agents.	[126]
	Nanotechnology-based drug delivery—targets anticancer agents specifically to leukemic cells, thereby reducing toxic side effects.	[131]
Pediatric Osteosarcoma	Lipid nanoparticles—methotrexate encapsulated in lipid nanoparticles is more effective for osteosarcoma treatment than free drug treatment.	[127]
Pediatric Brain Cancers	Nanoparticles—have potential as diagnostic tools and vectors for gene/drug therapy; targeting strategies to overcome the blood–brain barrier.	[128]
Children with Central Nervous System Tumors	Nanotechnology combined with proteomics—can help identify predictive biomarkers for metastatic spread.	[137]
Cholesteatoma Cells	Nanoparticle-based delivery of miR-34a—inhibits tumor cell proliferation and migration.	[129]
Pediatric Brain Tumor Cells	Nanoparticle-mediated delivery of siApe1—promising strategy for sensitizing cells to radiotherapy and circumventing resistance.	[130]
Pediatric Formulations	Nanotechnology development—have potential for greater therapeutic efficiency, drug targeting, reduced toxicity, and masking bitter drug taste, with anticancer drugs being the most commonly encountered therapeutic drug class.	[132]
Pediatric Solid Tumors	CLR1404 tumor-targeting radiopharmaceutical—benefits include selective uptake and prolonged retention, as well as potential for improved MRT treatment planning.	[138]
Ewing Sarcoma (EWS)	Nanomedicine Drug Delivery Systems—nanomedicine drug delivery systems offer promising alternatives for treating patients with recurrent or metastatic EWS, highlighting recent preclinical and clinical studies in epigenetics, immunotherapy, and nanotherapy, suggesting novel therapeutic strategies for EWS patients.	[139]
Pediatric Sarcomas	NanoString technology is a reliable approach for molecular diagnosis of pediatric sarcomas, detecting sarcoma-specific fusion transcripts in a single reaction with 100% concordance to RT-PCR. This study suggests future validation of additional sarcoma fusion transcripts and optimization of the workflow for diagnostic purposes.	[133]
Pediatric Ependymoma	High-resolution Mass Spectrometry proteomic analysis of pediatric ependymoma reveals similarities with other pediatric brain tumor entities, astrocytomas, and medulloblastomas. This study provides a basis for further research into ependymoma and its potential therapeutic targets.	[134]
Rhabdomyosarcoma	Thermosensitive liposome formulation with mild hyperthermia improved therapeutic index of vinorelbine.	[140]

**Table 2:** Nanosystems in pediatric Leukemia treatment.

Disease/Condition	Key Findings	Ref.
Leukemia	CHGNPs—a selective cytotoxic drug for clinical treatment via up-regulation of tumor suppressor protein P27 and inducement of G1 arrest.	[3]
Acute Lymphoblastic Leukemia (ALL)	Lipid-based Cubosomal Nanoformulations—combining metformin and cisplatin in lipid-based cubosomal nanoformulations may not be effective in treating ALL in children, highlighting the need for careful consideration when using nanotechnology to enhance drug efficacy.	[141]
	Gold Nanoparticle-based Nanocarriers—nanoparticle-based compounds have potential for drug delivery, cancer diagnosis, and therapy in ALL, providing an overview of conventional methods and nano-strategies for ALL treatment, with a special focus on gold nanoparticle-based nanocarriers of antileukemic drugs.	[4]
	Poly(lactide-co-glycolide) (PLGA) nanomedicines loaded with 6-mercaptopurine (6-MP) improve oral bioavailability and tissue distribution, leading to improve in vitro cytotoxicity of Jurkat cells and prolonged survival time in ALL model mice, demonstrating a promising delivery strategy for clinical translation.	[142]
	Nessler method using ultraviolet-visible spectrophotometry enables the quantification of PEGylated asparaginase activity in plasma for personalized nanomedicine in clinical settings.	[143]
B-cell Precursor Lymphoblastic Leukemia (BPL)	Polypeptide-based Nanoparticles—siRNA-mediated depletion of CD22DeltaE12 in BPL cells via polypeptide-based nanoparticles holds promise as a therapeutic strategy for high-risk and relapsed BPL patients.	[5]
B-cell acute lymphoblastic leukemia	NanoString nCounter technology—robust and cost-effective potential for B-ALL diagnosis with 100% sensitivity and 99% specificity.	[144]
Childhood Leukemia	Polymeric nanoparticles loaded with dexamethasone, enhance therapeutic efficacy, improving quality of life and survival.	[145]
Pediatric Acute Myeloid Leukemia	LNP-si-LINC01257 treatment using siRNA-loaded lipid nanoparticles, LNP-si-LINC01257 treatment using siRNA-loaded lipid nanoparticles is a safe and effective therapeutic approach for pediatric acute myeloid leukemia.	[6]

**Table 3:** Nanosystems in pediatric neuroblastoma treatment.

Disease/Condition	Key Findings	Ref.
Neuroblastoma	GASNGINAYLC peptide-coated nanovesicles have the potential for use in actively targeted neuroblastoma nanotherapy; are exceptional with regard to in vitro biocompatibility and stability.	[7]
	Peptide-functionalized liposomes—liposomes loaded with anticancer agents and functionalized with peptides enhance tumor-homing properties, induce tumor apoptosis, and reduce tumor glucose consumption, paving the way for novel targeted liposomal nanocarriers useful for multitargeting treatment of neuroblastoma.	[8]
	Manotechnology-based miRNA intervention shows promise in therapeutic management of neuroblastoma, overcoming challenges with drug delivery and enhancing therapeutic success.	[9]
	Liposomes—carriers for nano-drug delivery systems and specific drug targets, resulting in lower systemic side effects and improved pharmacokinetic properties of drugs.	[10]
	Nanomedicines have potential in relation to addressing the limitations of current diagnostic and therapeutic approaches, offering more effective and targeted options.	[11]
	Doxorubicin-loaded nanocarriers targeted at nucleolin—inhibition of cell proliferation, cell death, and tumor growth delay in vitro and in vivo.	[12]
High-risk neuroblastoma	Nanoparticle-based drug delivery systems with etoposide—synergy with alpha v integrin antagonists for improved patient care.	[13]
Neuroblastoma and Osteosarcoma	Amphiphilic antitumor agents—co-assembling two amphiphilic antitumor agents exhibit a better antitumor profile and controlled release behavior, providing a suitable pre-clinical candidate for childhood cancer therapy.	[14]
Anaplastic large cell lymphoma (ALCL)	Protamine nanomedicine with aptamers—dsDNA/drug payload, and siRNA, cell-selective chemotherapy and oncogene-specific gene therapy, targeting diagnostic biomarkers and therapeutic targets.	[146]
Neuroblastoma and medulloblastoma	Nanomedicines and cell-based therapies have the potential to reduce drug toxicity and improve efficacy, under investigation in phase I/II clinical trials.	[147]
Pediatric neuroblastoma	Nanomedicine has the potential to overcome the limitations of conventional chemotherapy.	[148]

**Table 4:** Nanosystems in pediatric osteosarcoma treatment.

Disease/Condition	Key Findings	Ref.
Multifocal osteosarcoma	Alpha-particle therapy with (227)Th and (223)Ra is a potential treatment for difficult-to-treat bone tumors due to its limited myelotoxicity and high relative biological effectiveness.	[15]
Osteosarcoma	Exosome mimetics derived from BMSCs Natural nano drug delivery platform with potent tumor inhibition activity and fewer side effects.	[16]
	Nanocarriers present a promising opportunity to improve treatment efficacy and reduce side effects.	[17]
	Targeted drug delivery systems have the potential to overcome drug resistance and reduce side effects.	[18]
	Edelfosine-loaded lipid nanoparticles effectively decrease cell growth in vitro and prevent metastasis in vivo, showing promise for chemotherapy.	[19]
	Micelleplexes for Nucleic Acid Delivery and Active Targeting Nanotechnology offers new avenues for selective targeting of osteosarcoma, with a focus on ligand-mediated strategies. Future directions for osteosarcoma diagnosis and therapy using nanotechnology are also discussed.	[149]
	Nanocarrier Exosomes hold promise as a nanocarrier for treating osteosarcoma, summarizing recent research on using exosomes as a therapeutic approach and highlighting their ability to mediate intercellular communication while reducing toxicity.	[150]
	Biodegradable and CO_2_-derivative cationic poly(vinylcyclohexene carbonates). Significant tumor regression both in vitro and in vivo.	[151]
	Photodynamic therapy (PDT) has potential as a minimally invasive treatment for deep tumors, with recent developments and novel strategies showing promise.	[152]
	Near-infrared imaging and multifunctional graphene-based nano-drug delivery system. Highly selective anticancer efficiency in targeting mitochondria and demonstrating synergistic phototherapy for drug-resistant osteosarcoma.	[21]
	Self-stabilized hyaluronate nanogel co-delivers doxorubicin and cisplatin Enhanced antitumor efficacy and reduced side effects through prolonged circulation and synergistic apoptosis induction, showing great potential for osteosarcoma chemotherapy.	[20]
	IL-11Ralpha-targeted nanoparticles have greater efficacy in treating osteosarcoma, specifically targeting tumor cells, and have strong anti-tumor effects in orthotopic and relapsed OS models and patient-derived OS xenografts.	[22]
	Lipid-based nanoparticles show potential in treating osteosarcoma, specifically targeting tumor cells and reducing systemic toxicity; different lipid nanocarriers have the potential to deliver anti-osteosarcoma drugs.	[153]

**Table 5:** Nanosystems in other pediatric cancer treatments.

Disease/Condition	Key Findings	Ref.
Pediatric brain tumors	Nanotechnology has potential to enhance drug delivery across the blood–brain barrier.	[154]
Diffuse intrinsic pontine glioma (DIPG) and other brain tumors	Polymeric nanoparticles surface-modified with a protease-resistant peptide shuttle have potential benefits in treating brain tumors with a fully conserved blood–brain barrier.	[155]
Brain Tumors	Hydrogen sulfide (H(2)S) and RNA-based nano-delivery—potential to improve therapeutic results for pediatric primary brain tumors. The lack of available treatments due to the blood–brain barrier necessitates a full understanding of molecular pathways.	[156]
Medulloblastoma	Nanotechnology—overcoming delivery challenges in using noncoding RNAs as therapeutic targets for medulloblastoma, a type of childhood brain tumor.	[157]
	Nano preparation of phytoconstituents incorporate plant products into nanocarriers, enhancing bioavailability for medulloblastoma treatment.	[158]
Medulloblastoma	Nanotechnology-based solutions show potential for overcoming the blood–brain barrier in medulloblastoma treatment and can selectively target pediatric brain tumors.	[159]
Diffuse Intrinsic Pontine Gliomas	Nanoparticles, including those that can cross the blood–brain barrier and theranostic nanoparticles, hold potential for the treatment of DIPG, and more attention should be directed towards developing a nanoparticle delivery system specifically for DIPG treatment.	[160]
Retinoblastoma	Nanodelivery systems overcome the side-effects and reduced efficacy of traditional chemotherapy, with sustained drug release and targeted drug delivery to the site of the tumor, offering a high survival rate for this rare pediatric cancer in low- and middle-income countries.	[164]
	Photodynamic therapy (PDT) using mesoporous silica nanoparticles (MSN) shows potential for treating retinoblastoma and highlights the role of nanomedicine in developing effective treatments for ophthalmological purposes.	[161]
Cancer and Regenerative Medicine	Nanomedicine-based therapies show promise in cancer and regenerative medicine, as well as drug delivery systems for brain and cardiac repair. Combining stem cells with drug delivery systems shows improved results.	[162]
Human Cancers	Green nanotechnology for nano radiopharmaceuticals has potential in the effective diagnosis and treatment of cancers.	[165]
Primary mitochondrial disorders (PMDs)	Nanocarrier-based treatments—effective treatments, especially in children, possible with nanotechnology.	[166]
Rhabdomyosarcoma	Alpha-Fe_2_O_3_ and SiO_2_ nanoparticles induce apoptosis in rhabdomyosarcoma cells and provide potential insights into the mechanism of cell death. Further investigation at the in vivo level is needed.	[167]
Childhood Malignancies	CD19-targeted delivery of doxorubicin encapsulated in polymeric nanoparticles reduces treatment-related side effects.	[168]
Brain Cancer	Survivin-targeting treatments and nanomedicine—nanoparticle technology has the potential to overcome the blood–brain barrier and deliver therapeutic effects for brain cancer therapy.	[169]
Adenoid cystic carcinoma (ACC)	Gold nanoparticles bound to purified polyclonal antibody have the potential for tumor diagnostics via imaging or as a delivery device for targeted therapy.	[163]
Oncofertility	Pre-pubertal ovarian tissue nanotechnology research raises ethical questions about the generation of “NUBorn” and “NUAge” mice but argues for the moral permissibility and necessity of the work, with justice and vulnerable subjects’ protection as central considerations.	[170]

**Table 6:** Nanosystems in pediatric malaria and other bacterial diseases.

Disease/Condition	Key Findings	Ref.
Malaria	Nanomedicines offer a promising approach for targeted drug delivery in the treatment of malaria. Various nanotechnology-based strategies provide tools to design strategies for targeting drug molecules to specific stages of the malaria parasite, treating drug-resistant parasites, increasing vaccine efficacies, and more.	[23,24]
Leishmaniasis	New drug delivery systems using nanotechnology aim to decrease toxicity of amphotericin B while maintaining therapeutic efficacy.	[25]
Pediatric diseases (malaria, leishmaniasis, toxoplasmosis, and schistosomiasis)	Nanotechnology-based drug delivery systems can provide innovative solutions for challenges in administering drugs to pediatric patients.	[171]
Schistosomiasis	The use of nanoparticles in bioassays has the potential to provide higher sensitivity, rapidity, and convenience for the detection and control of schistosomiasis, contributing to the improvement of public health.	[26]
Helminthiasis	Nanocarriers for Praziquantel (PZQ) offer a promising approach to improve PZQ performance by overcoming low solubility and bioavailability limitations, with recent advances in PZQ nanoformulations showing improved solubility and bioavailability.	[27]
Bacterial Infections in Human Kidneys	A biosensor using nano-fabricated structures and anti-E. coli antibodies shows potential for clinical use in detecting bacterial infections in human kidneys with high sensitivity.	[29]
Antibiotic Resistance	Chitosan nanoparticles enhance the antimicrobial efficiency of ceftriaxone against Gram-positive and Gram-positive bacteria and can be an alternative to combat antibiotic resistance.	[28]
Pediatric infectious diseases and solid tumors	Nanotechnology shows potential for treatment, from in vitro to clinical trials.	[172]

**Table 7:** Nanosystems in pediatric COVID-19 treatment.

Disease/Condition	Key Findings	Ref.
SARS-CoV-2	Diagnostic methods optimization using nanotechnology. Advancements in COVID-19 detection, including CRISPR-based and optical-based sensing systems.	[173]
	A liposome-mediated detection method accurately detects SARS-CoV-2 RNA-positive extracellular vesicles in plasma, providing a viable diagnostic alternative to respiratory RNA levels with promising clinical characteristics.	[174]
COVID-19	Nanotechnology and AI for diagnosis and treatment options; advancements in diagnosis and treatment options, vaccination, and potential for nanotechnology and AI.	[175]
Newborn screening	A novel electrochemical nano-biosensor based on signal amplification using nanomaterials can detect harmful DNA mutations in newborn children with a high detection limit, wide dynamic range, and great specificity.	[176]

**Table 8:** Nanosystems in pediatric TB and HIV treatments.

Disease/Condition	Key Findings	Ref.
TB and HIV	Nanomedicine for antimicrobial host defense peptide delivery. Nanomedicine enhances therapeutic efficacy and reduces resistance.	[30]
HIV diagnosis in infants	Nanotechnology-based diagnostic methods for accurate HIV diagnosis. Nanotechnology-based methods show promise for detecting HIV in infants.	[31]
HIV	Nanotechnology-based efavirenz liquid formulation could lead to improved bioavailability and show promising absorption profiles, making it an effective dose-adjustable treatment for pediatric patients.	[32]
Antiretroviral drug formulations	New formulations of antiretroviral drugs using nanomedicine can increase bioavailability and reduce dose.	[33]
Pediatric HIV-1 infection	Nanotechnology-based drug delivery systems and computer-aided drug design present promising solutions for improving antiretroviral therapy efficacy, which faces challenges such as drug resistance and inefficient viral reservoir targeting.	[34]
Tuberculosis	Nanotechnology-based antigen testing has a high diagnostic accuracy for early TB detection and monitoring anti-TB treatment responses in HIV-exposed infants.	[35]
	“Flower-like” polymeric micelles. Polymeric micelles increase oral bioavailability of rifampicin, making them a potential platform for developing an extemporaneous liquid fixed-dose combination with isoniazid for pediatric administration to treat tuberculosis.	[36]
	Nanotechnology can potentially address the challenges associated with the treatment of tuberculosis by developing more effective and compliant medicines, overcoming drug resistance, reducing treatment length, and targeting infection reservoirs.	[37]
	Nanomedicine and genomic research for novel antituberculous therapeutics. Potential for achieving tuberculosis elimination by 2050, but requires greater institutional support.	[38]
	Child-friendly nanoemulsion for tuberculosis treatment—nanoemulsion-containing rifampicin increases drug bioavailability and reduces treatment failure.	[39]

**Table 9:** Nanosystems in pediatric respiratory and pulmonary diseases.

Disease/Condition	Key Findings	Ref.
Sepsis	Nanoparticle-based technologies show potential in sepsis detection and management.	[177]
Ventilator-associated pneumonia (VAP) in pediatric patients	Nanoparticles prevent biofilm formation and colonization on endotracheal tubes, reducing VAP risk.	[40]
	Nanomodified endotracheal tubes effectively combat VAP, as demonstrated by a 2.7 log reduction in *P. aeruginosa* growth on nanoroughened ETTs.	[41]
Respiratory Syncytial Virus (RSV)	Nanotechnology has the potential to detect and therapeutically treat Respiratory Syncytial Virus with maximal therapeutic efficacy and minimal side effects.	[42]
	Gold nanorods can inhibit RSV through immune response activation, making them a potential antiviral agent against RSV.	[43]
Cystic fibrosis	The Nanoduct sweat test system is easier and has a higher success rate in diagnosing cystic fibrosis in newborns than Macroduct/Gibson and Cooke methods.	[44]
Cystic fibrosis pain	Nanotechnology-based approaches show promise in managing cystic fibrosis pain, a common disease-related pain for CF patients.	[45]

**Table 10:** Environmental health and pediatric infectious diseases.

Disease/Condition	Key Findings	Ref.
Placental transfer of nanoparticles	Ex vivo human placental perfusion model for nanoparticle transfer. Human placental perfusion model reveals transfer of nanoparticles across placenta, highlighting potential risks.	[46]
Water-borne protozoan parasites	Biosensors and nanotechnology-based detection methods offer more reliable and efficient detection in the environment.	[47]
Drinking water contamination	Nanotechnology-based treatments present effective solutions for removing biological contamination, particularly in developing countries with poor sanitation.	[48]
Waterborne pathogens	Nanoparticle-based assays and nanodevices have been introduced as potential solutions to microbial detection challenges in detecting water-borne pathogens for the purpose of safeguarding public health.	[49]
Environmental health issues	Coordinated efforts are needed to limit the potential consequences of emerging environmental health issues in the Pacific Basin region, particularly on children’s health, caused by climate change and increasing production of environmental pollutants.	[178]
Scorpion envenomation	Biocompatible nanoparticles as specific vectors for antigen-presenting cells. Nanotechnology provides a potential solution to the life-threatening nature of scorpion envenomation by inducing active, protective immunity against venom toxins.	[50]
Measles virus	Gold nanoparticles synthesized using natural extracts demonstrate promising antiviral activity and present a potential strategy for treating and controlling viral infections.	[51]
Human metapneumovirus	Nanotechnology and advanced genetic platforms—PCR-based assays remain most reliable, exploring potential benefits for therapeutic intervention.	[52]

**Table 11:** Nanosystems in medical treatments.

Disease/Condition	Key Findings	Ref.
Epilepsy	Glutathione pegylated liposomal methylprednisolone for potential sustained drug delivery system using liposome nanotechnology.	[53]
Inborn errors of metabolism	Mass spectrometry using nanotechnology and genomics advancements can facilitate cost-effective expanded newborn screening for detecting more disorders.	[54]
Cardiovascular disease and cancers	Saliva as a diagnostic fluid—potential for noninvasive, cost-effective, and gland-specific detection, with promising future in nanotechnology despite current clinical barriers.	[55]
Traumatic brain injury	Core-cross-linked nanoparticles reduce neuroinflammation and the secondary spread of injury in a mouse model—potential strategy for treatment.	[56]
Neurodegenerative diseases	Targeted drug delivery and monitoring. Nanobiotechnology improves bioavailability and reduces side effects for Parkinson’s, Alzheimer’s, multiple sclerosis, amyotrophic lateral sclerosis, Huntington’s, and Wilson’s diseases.	[57]
Gestational diabetes and jaundice	Nanotechnology and biosensors, as well as appropriate biomarkers and serum-based biomarkers, have the potential for high-performance diagnosis, emphasizing the need for monitoring during pregnancy.	[58]
Osteogenesis Imperfecta	A nanoindentation study found isotropic properties in type III Osteogenesis Imperfecta bone tissue, regardless of age, with the potential to aid diagnosis and treatment.	[59]
	Nanotechnology-assisted diagnosis and treatment—similar mechanical properties found in type III and type IV bone tissue, aiding diagnosis and treatment.	[60]
Hereditary Angioedema	Nanofiltered C1 Esterase Inhibitor—preprocedural administration of nanofiltered C1 esterase inhibitor effectively prevents hereditary angioedema attacks during dental, medical, or surgical procedures, with no reported adverse events.	[61]
Aedes Aegypti Mosquito	Polymeric nanocapsules containing geraniol and icaridin are effective and safe against the Aedes aegypti mosquito—a leading disease transmitter.	[62]
Bone formation and microstructure	Alendronate treatment promotes bone formation with a less anisotropic microstructure during intramembranous ossification in rats but may deteriorate the material properties of the bone microstructure.	[179]
Tissue-engineered vascular grafts	Nanotechnology-assisted growth and inflammation pathway insights offer wide clinical applications with potential benefits for second-generation grafts.	[180]
Micronutrient deficiency	Nanoparticle coating for vitamin B(12) fortification enhances the bioavailability of vitamin B(12) in food crops, reducing the risk of micronutrient deficiency and its associated health risks.	[63]
Vulvovaginitis in Girls	Nanotechnology-based treatment options: Bioyoghurt, probiotics, and petroleum jelly are useful for managing vulvovaginitis in girls.	[64]
Invasive Surgeries	The integration of nanotechnology with pure sciences and fourth industrial revolution technologies shows potential for the development of a nano-doctor, eliminating the need for invasive surgeries and benefiting human health.	[65]
Children’s health	Nanobiology and nanomedicine provide promising implications for children’s health and show potential for innovative nanodevices combining diagnostics and therapeutics.	[66]
Pediatric diseases	Albumin and liposome nanoparticles show great potential for treatment.	[67]
T-cell immunology	Microengraving technology–efficient identification of antigen-specific T-cell responses.	[68]
Pediatric drug development	Nanotechnology-based delivery systems have the potential to address the lack of pharmacokinetic data.	[69]
Traumatic Brain Injury (TBI)	Nanoparticle-based delivery strategy—overcomes biological barriers and increases target engagement for effective treatment of TBI.	[70]
Congenital Bladder Dysfunction	Nanoparticle-based tissue engineering—potential drug delivery system and creation of de novo scaffolds for developing urinary bladders.	[71]
Pediatric Urological Conditions	Nanotechnology-based tissue engineering and stem cell therapy—promising solutions for regenerating kidney, bladder, and urethra tissues.	[181]
Pregnancy complications	Nanomedicine-based therapies have the potential to treat pregnancy complications, improving prognosis for mothers and unborn children.	[72]
Cerebral palsy	Personalized nanomedicine—gold nanoparticles coated with targeted dendrimer can improve motor function in children with cerebral palsy.	[73]
	Nanomedicine and stem cell therapy—diagnosis and treatment approaches for high-risk factors in cerebral palsy, future research directions suggested.	[74]
Hereditary angioedema	Nanofiltered human C1 inhibitor concentrate (C1-INH NF)—safe and effective replacement for deficient plasma C1 inhibitor levels.	[75]
Scoliosis	Ultra-low-dose full-spine protocol using nanotechnology—good reliability and repeatability for reproducible Cobb angle measurements.	[76]
Fetal, neonatal, and pediatric diseases	Diagnostic and therapeutic applications—promising for respiratory tract, neurosensory system, and infections.	[77]

**Table 12:** Nanosystems in dental diseases.

Disease/Condition	Key Findings	Ref.
Children’s occlusal cavities	Resin coating—nanotechnology improves wear resistance of dental materials.	[78]
Dental Plaque and Gingival Inflammation	Oral spray containing resveratrol delivered via nanovectors—reduces dental plaque and gingival inflammation in early childhood.	[79]
Pediatric Dentistry	Nanoparticles—potential benefits and drawbacks of nanoparticles in pediatric dentistry require further study.	[83]
	Nanotechnology—advances in procedures, materials, and techniques, including nanotechnology, in pediatric dentistry.	[182]
	Polysaccharide-based drug delivery systems show great potential for preventing and treating oral diseases in pediatric patients.	[80]
	Silver nanoparticles (AgNPs) enhance the antimicrobial and mechanical properties of dental materials in pediatric dentistry.	[81]
Dental Sealants	Nanoparticles of amorphous calcium phosphate create antibacterial and rechargeable sealants for dental applications.	[183]
Pediatric Laryngotracheal Stenosis	Magnesium-alloy based airway stents—feasible and effective management technique for pediatric airway obstruction.	[82]

**Table 13:** Nanosystems in pediatric dermatologic diseases.

Disease/Condition	Key Findings	Ref.
Atopic dermatitis	Nanocarriers for drug delivery improve drug solubility, thermodynamic activity, and skin permeation, reducing side effects and managing atopic dermatitis effectively.	[84]
	Nanomaterials present effective drug delivery solutions.	[85]
	Chitosan nanoparticles (CS-NPs) for drug delivery—encapsulating betamethasone valerate into CS-NPs improves drug penetration and efficacy for atopic dermatitis.	[86]
	Nanocarrier-mediated drug delivery—recent clinical evidence and recommendations support the use of nanocarrier-mediated drug delivery for treating atopic dermatitis.	[185]
Acne	Nanotechnological carriers show great potential in improving the efficacy and safety of anti-acne drugs, though further progress is needed.	[184]
Neonatal Scleredema	Polydopamine nanoparticles for inhibiting fibrosis in neonatal scleredema—polydopamine nanoparticles coated with stem cell membrane fragments and doxorubicin inhibit fibrosis.	[87]

**Table 14:** Nanosystems in pediatric nutrition.

Disease/Condition	Key Findings	Ref.
Obesity	Multidisciplinary collaborations between engineers, physical scientists, and obesity and nutrition experts can develop useful technologies to support therapeutic advances and behavioral change to address the obesity epidemic.	[88]
Nutritional deficiencies, obesity, and type 2 diabetes	Nanotechnology-based food production—nanotechnology has potential in producing more nutritious and low-calorie foods to control nutritional deficiencies, obesity, and type 2 diabetes. Collaboration between food producers, policy makers, and health authorities is needed to address safety concerns and fund research in this area.	[89]
Iron deficiency	Iron solid lipid nanoparticles (Fe-SLNs)—Fe-SLNs with enhanced bioavailability are promising for iron supplementation, overcoming limitations of commercially available supplements.	[90]
Iron supplementation	Green-synthesized iron oxide nanoparticles (IONPs)—low doses of green-synthesized IONPs show potential as antianemic preparations, while high doses cause toxicity and histopathological alterations in major organs in rats.	[186]
Food safety	Optical biosensors using gold nanoclusters (AuNCs)—surface-functionalized AuNCs can serve as optical biosensors for food safety, detecting harmful pathogens and chemical substances. The review summarizes the recent progress of AuNCs as optical biosensors and their application in food safety.	[91]
Food allergies and anaphylaxis	Nanotechnology-based diagnostic methods can enable personalized allergen immunotherapy and avoidance diets to address the increasing prevalence of food allergies and anaphylaxis.	[92]

**Table 15:** Nanosystems in pediatric drug delivery.

Disease/Condition	Key Findings	Ref.
Micronutrient delivery	Biomimetic nanovesicles impregnated in transdermal patches, used for the transdermal delivery of folic acid and iron, show promising results in improving micronutrient delivery in infants and mothers.	[93]
Poorly water-soluble drugs	Lipid-based nanotechnology with in situ self-assembly nanoparticles yields improved stability, palatability, and bioavailability of pediatric oral solid dosage forms.	[94]
	Electrospun polyvinyl alcohol (PVA) nanofiber films yield improved biopharmaceutical properties for the oromucosal administration of drug-loaded nanofiber films for pediatric or geriatric patients.	[99]
	In situ self-assembly nanoparticle technology provides a promising platform for drug delivery.	[95]
Drug delivery	Folic acid magnetic nanotheranostics hold the potential for safe and effective drug delivery, reducing cardiotoxicity and promoting drug uptake.	[96]
	Nanoparticle-based drug delivery system—a novel methodology produces 400 micron spheroids suitable for easy consumption, with potential to add drugs, benefiting patient compliance.	[97]
Oromucosal drug delivery	Electrospun polycaprolactone nanofibers provide a promising approach for drug delivery in children and the elderly.	[98]
Pediatric medication	Spironolactone-loaded nanocapsules provide a promising approach with stable nanocapsules and high encapsulation efficiency, enabling lower volumes of liquid preparation.	[100]
Taste masking in oral drugs	Nanotechnology has the potential for improved taste-masking properties.	[187]
Vaccine delivery	Nanopatch—high acceptability for vaccine delivery in low and middle-income countries.	[101]
Cosmetics and nanocosmeceuticals	Gallic acid and its derivatives—poor water solubility and biodegradability require nanoformulations.	[102]

**Table 16:** Nanosystems in other pediatric disorders.

Disease/Condition	Key Findings	Ref.
Sleep-related disorders	LC-MS/MS assays for melatonin and N-acetylserotonin—highly sensitive assays at pg/mL levels show potential as biomarkers for sleep-related disorders.	[188]
Profound deafness	Cochlear implants—nanotechnology research could lead to high-fidelity sound and implants in ears with useful hearing.	[189]
Chronic tonsillitis and other diseases	Metallic particles in human tonsil tissue and amniotic fluid could help to point towards alternative causes of some diseases and emerging nanopathology.	[190]
Exposure to nanoparticles	Nano-enabled consumer products—resuspension rates of particles vary depending on the product, flooring, and resuspension force. Products containing Cu, Ag, and Zn nanomaterials had higher rates.	[191]
Pediatric degenerative ocular diseases	Ocular nanomedicines—recent developments in drug delivery systems and nanomedicines show promise for treating degenerative ocular diseases with childhood onset.	[192]
Microbial interaction	Glucosyltransferase B (GtfB) and Candida albicans—streptococcus mutans-derived exoenzyme GtfB binds strongly to Candida albicans, explaining how it modulates this virulent cross-kingdom interaction.	[193]
Mercury contamination	Nano-selenium reactive barriers suppress mercury release from compact fluorescent lamps, indicating the location of Hg contamination and achieving significant suppression of mercury release in three exposure prevention scenarios.	[194]
On-site monitoring of Pb(II)	Gold nanoparticle-based dynamic light scattering (DLS) probe can detect Pb(II) at levels as low as 100 ppt (almost two orders of magnitude higher than the EPA standard limit) and has potential applications for on-site monitoring of Pb(II) in various samples.	[195]

**Table 17:** Nanosystems and their potential risks and impact on pediatric health.

Disease/Condition	Key Findings	Ref.
General safety rules for nanotechnology	Graphene is a safer option compared to carbon nanotubes in nanotechnology. Specific safety rules must be implemented, including the use of small sheets and hydrophilic dispersions, and safety risks should not be generalized.	[199]
Children’s health and exposure to nanoparticles	Engineered nanoparticles—monitoring of the impact of engineered nanoparticles (ENPs) on children’s health is necessary. Techniques are needed to determine toxic effects and exposure levels to ensure the safety of all nanoparticle-based products.	[103]
Impact of nanoparticles on breastfeeding	Nanoparticles can cause metabolic burden, oxidative stress, and altered milk composition, indicating potential risks to the breastfeeding system. Specific proteins modified by S-glutathionylation by nanoparticles provide insight into molecular mechanisms of nanotoxicity.	[196]
Neurotoxicity of nanoparticles	Combustion and friction-derived nanoparticles, industrial nanoparticles, and nanomedicine—nanoparticles can cause neurovascular unit and organelle damage and protein misfolding. Nanoparticle exposure carries a high risk for the developing brain homeostasis and should be included in Alzheimer’s and Parkinson’s disease research framework.	[104]
Health effects of carbon nanoparticles	Carbon nanoparticles—more research is needed on the potential health effects of carbon nanoparticles on children’s health and whether they act as nano-vectors of other carcinogenic pollutants.	[106]
Children’s exposure to bioavailable silver	Nanotechnology-based consumer products have limited potential for children’s exposure to bioavailable silver in ionic rather than particulate form, despite silver being released mainly through dissolution in high salt concentrations.	[200]
Neurotoxicity of metal nanoparticles	Engineered metal nanoparticles can induce greater neurotoxicity in young and elderly rats, with small-sized NPs and composition affecting blood–brain barrier breakdown, brain edema, neuronal injuries, and myelin vesiculation.	[105]
Toxicity and inflammogenicity of ultrafine particles	Ultrafine particles demonstrate increased toxicity and inflammogenicity, highlighting the need to study diverse nanoparticle compositions and structures to determine their extent of toxicity and altered properties within nano dimensions and to further investigate their impact on health and the environment.	[107]
Children’s vulnerability to nanoparticles	Engineered nanoparticles pose potential risks to infants and children, highlighting the need for focused studies on exposure levels and health consequences. Future research and regulations in ENP applications are necessary to understand the potential benefits of nanotechnology.	[197]
Public understanding of nanotechnology	Nanotechnology in society—public understanding of nanotechnology is lacking, especially among middle-school children, despite its importance in industry and society. A survey revealed the need for a firm foundation of understanding nanotechnology’s context and potential benefits in the world that is too small to see.	[198]
Impact of ultrafine particulate matter on children’s health	Ultrafine particulate matter—children are vulnerable to harmful effects induced by exposure to ultrafine particulate matter. Research is needed to understand the differences between children and adults in this regard, but nanoparticles’ unique physico-chemical properties offer promising new possibilities for biomedical applications. The potential benefits of nanoparticles in nanotechnology should be explored.	[108]
